# Hydroxychloroquine/chloroquine in patients with COVID-19 in Wuhan, China: a retrospective cohort study

**DOI:** 10.1186/s12879-021-06477-x

**Published:** 2021-08-12

**Authors:** Zhe Chen, Aihua Liu, Yongjing Cheng, Xutao Wang, Xiaomao Xu, Jia Huang, Yuqing Ma, Ming Gao, Cibo Huang

**Affiliations:** 1 Department of Rheumatology, Beijing Hospital, National Center of Gerontology, Beijing, China; 2 Department of Emergency, Beijing Hospital, National Center of Gerontology, Beijing, China; 3 Department of Pulmonary and Critical Care Medicine, Beijing Hospital, National Center of Gerontology, Beijing, China

**Keywords:** Hydroxychloroquine, Chloroquine, Virus shedding periods, COVID-19

## Abstract

**Background:**

Since the COVID-19 pandemic, several therapeutic agents have been used in COVID-19 management. However, the results were controversial. Here, we aimed to evaluate the efficacy and safety of hydroxychloroquine (HCQ)/chloroquine (CQ) in COVID-19.

**Methods:**

We retrospectively reviewed the medical charts of patients with COVID-19 admitted to an inpatient ward in Wuhan from 2020/Feb/08 to 2020/Mar/05. Patients with HCQ/CQ and age, gender, disease severity matched ones without HCQ/CQ were selected at a 1:2 ratio. The clinical, laboratory and imaging findings were compared between these two groups. The multivariate linear regression analysis was performed to identify the factors that might influence patients’ virus shedding periods (VSPs).

**Results:**

A total of 14 patients with HCQ/CQ and 21 matched ones were analyzed. The HCQ/CQ treatment lasted for an average of 10.36 ± 3.12 days. The mean VSPs were longer in the HCQ/CQ treatment group (26.57 ± 10.35 days vs. 19.10 ± 7.80 days, *P* = 0.020). There were 3 patients deceased during inpatient period, two patients were with HCQ/CQ treatment (*P* = 0.551). In the multivariate linear regression analysis, disease durations at admission (t = 3.643, *P* = 0.001) and HCQ/CQ treatment (t = 2.637, *P* = 0.013) were independent parameters for patients’ VSPs. One patient with CQ had recurrent first-degree atrioventricular block (AVB) and obvious QTc elongation, another one complained about dizziness and blurred vision which disappeared after CQ discontinuation. One patient with HCQ had transient AVB.

**Conclusions:**

In summary, we identify that the HCQ/CQ administration is not related to less mortality cases at later phase of COVID-19. More studies are needed to explore whether HCQ/CQ treatment would lead to SARS-Cov-2 RNA clearance delay or not.

**Supplementary Information:**

The online version contains supplementary material available at 10.1186/s12879-021-06477-x.

## Background

Since December 2019, the outbreak of severe acute respiratory syndrome coronavirus 2 (SARS-Cov-2) infection has swept over the whole world in a few months. By May 5, 2020, more than 3.5 million cases have been confirmed and the death toll raises to over 250 thousand all around the world. The SARS-Cov-2 infection results in the coronavirus disease-2019 (COVID-19), which is composed of a spectrum of clinical manifestations including pneumonia, heart/kidney/liver injury, and coagulopathy, etc. [1, 2].

Due to lacking of specific anti-virus drugs, the management of COVID-19 is still challenging. The results of two randomized controlled clinical trials of the promising anti-virus agents, i.e. Lopinavir/Ritonavir and Remdesivir, showed that these drugs were not that effective as we had expected in Chinese patients with COVID-19 [3, 4]. Because of the dramatic elevation of several inflammatory factors, such as interferon-γ-induced protein 10, monocyte chemotactic protein-3, interleukin-13, et al*.*, overreactive immunopathological mechanisms were surmised to be responsible for multiple organ damage in COVID-19 [5]. Some researchers hypothesized that patients with COVID-19 might benefit from some anti-rheumatic drugs, such as hydroxychloroquine (HCQ)/chloroquine (CQ) and tocilizumab (TCZ), for their dual effects on immune regulation and suppression [6].

CQ is a traditional anti-malaria drug. As a derivate of CQ, HCQ is less toxic to retina and heart and is the background treatment in systemic lupus erythematosus (SLE) [7]. Yu and colleagues reported that HCQ treatment could reduce serum interleukin-6 (IL-6) levels in COVID-19 patients [8]. Except for the anti-inflammatory activity, HCQ/CQ has potential anti-virus effects [9, 10]. Liu et al. reported that both HCQ and CQ could inhibit SARS-Cov-2 replication and prevent the virus from entering into cells in vitro [11]. HCQ/CQ was recommended as an option in the COVID-19 management guideline in China [12, 13]. Furthermore, both the US Food and Drug Administration and the Indian Council for Medical Research had permitted the empiric use of HCQ in COVID-19 patients [14, 15]. With the surging demands for HCQ in COVID-19, some patients under long-term HCQ treatment for autoimmune disease, such as SLE, were threatened by HCQ shortage. Therefore, some rheumatologists campaigned for using HCQ rationally in COVID-19 in which the data and evidence were limited and inconclusive [16]. Unfortunately, the efficacy of HCQ/CQ in COVID-19 remained equivocal by far.

At the very beginning of the outbreak of COVID-19, a multidisciplinary medical team from Beijing Hospital took in charge of an independent inpatient ward to manage the COVID-19 patients in the Sino-French New City Branch of Tongji Hospital in Wuhan, China. Some patients took HCQ/CQ during their inpatient period. We performed the following retrospective analysis to evaluate the potential efficacy and safety of HCQ/CQ in COVID-19.

## Methods

### Patients

Medical charts of patients admitted to one inpatient ward in Wuhan from February 08, 2020 to March 05, 2020 were reviewed. Due to the potential while uncertain efficacy of TCZ in COVID-19, patients receiving TCZ treatment were excluded from the study. Patients with HCQ/CQ treatment and age, gender, disease severity matched ones without HCQ/CQ treatment were analyzed. The matching process was performed with the SPSS software (version 26.0) and the propensity-score (PS) matching package at a 1:2 ratio. The Caliper value was 0.2.

### Methods

This was a retrospective cohort study. The demographic data, clinical manifestations, comorbidities, laboratory findings and image involvement patterns assessed by computed tomography (CT) were carefully and thoroughly collected from medical charts.

The disease severity was defined as mild, general, severe and critically severe according to the Chinese management guideline for COVID-19 (Additional file 1) [12]. The CURB-65 severity score was calculated according to the standard definition [17]. The estimated glomerular filtration rate (eGFR) was calculated via the CKD-EPI equation [18]. The concurrent respiratory pathogen infections, including type A influenza, type B influenza, mycoplasma pneumoniae, chlamydia pneumoniae, respiratory syncytial virus, adenovirus, parainfluenza virus and legionella pneumophilia infections, were confirmed by the presence of pathogen specific immunoglobulin M with the enzyme-linked immunosorbent assay.

The nasopharyngeal swabs were sampled based on physicians’ judgement on clinical purposes. And the ribonucleic acids (RNAs) of SARS-Cov-2 were examined with the polymerase chain reaction (PCR) method [19]. The virus shedding periods (VSPs) were defined from symptoms onset to the first day of the consecutive negative PCR results before discharge (Additional file 1). Drugs taken by the patients for COVID-19 management purposes before admission were recorded and analyzed as well. Receiving corticosteroids (GCs) treatment was defined as exposure to systemic GCs. The dosage of GCs was calculated by methylprednisolone (MP) (prednisone:methylprednisolone = 1.25:1). The complains and symptoms after HCQ/CQ initiation were carefully recorded.

### Statistical analysis

Statistics analyses were conducted with the SPSS software (version 26.0). Numerical data was expressed as mean ± standard deviation (SD) or quartiles (Q1: first quartile; Q2: second quartile; Q3: third quartile), while categorical data was expressed as numbers and percentages. Numerical data was compared with the independent sample *t*-test. Categorical data was compared with the Chi-square or the Fisher’s exact test, as appropriate. The multivariate linear regression analysis was performed to identify the factors that might influence patients’ VSPs. Virus shedding periods were the dependent variable. Continuous or dichotomous parameters, such as disease duration at admission, with or without HCQ/CQ treatment, dosage of GCs et al*.* selected according to clinical judgment, were analyzed as probable predict variables with the stepwise method in the multivariate linear regression analysis (Additional file 1: Table S1). Laboratory results which were statistically different between patients with and without HCQ/CQ were selected as probable predict variables as well. MP dosage in patients without GCs was recorded as zero. All probabilities were 2-sided, and P values < 0.05 were considered to be statistically significant.

## Results

**Fig. 1:**
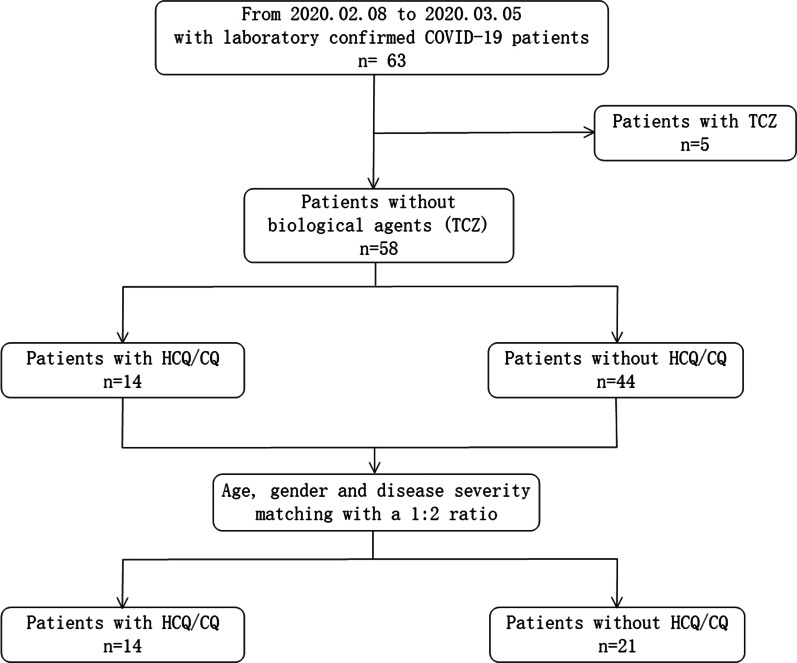
The flow diagram of patient selection in the present study. *COVID-19* corona virus disease-2019, *TCZ* tocilizumab, *HCQ* hydroxychloroquine, *CQ* chloroquine

From February 8, 2020 to March 5, 2020, a total of 63 COVID-19 patients were admitted to our ward. All the patients were confirmed with SARS-Cov-2 infection via PCR. Except for 5 patients who received TCZ treatment, 58 patients were treated by non-biological drugs. Among the 58 patients, 11 and 3 patients received HCQ and CQ treatment, respectively. After age, gender and disease severity matching, 21 patients without HCQ/CQ treatment were selected as controls and were further analyzed (Fig. 1).

For the 35 patients, the average age was 62.20 ± 11.88 years old with a male predominance. The span from symptoms onset to admission were 13.00 ± 7.24 days. Although common at disease onset (77.14%), fever was observed in only 20% of patients at admission. Twenty-six patients (74.28%) had at least a comorbidity, most of which was hypertension. Twenty-one out of the 35 patients suffered from multiple pathogen infections in addition to SARS-Cov-2. And influenza was the most common concomitant infectious disease (57.14%) (Table 1). Procalcitonin elevation was recorded in 9 patients. Serum ferritin and IL-6 levels were elevated in 34 and 17 patients, respectively. Serum IL-1β, IL-2R, IL-8, IL-10 and tumor necrosis factor-α (TNF-α) levels were tested in 14 patients. As a result, serum IL-1β, IL-2R, IL-8, IL-10 and TNF-α elevation were recorded in 1, 8, 2, 0 and 6 patients, respectively.

**Table 1:** Clinical characteristics, laboratory and imaging findings of the 35 patients at admission

	Total (n = 35)		With HCQ/CQ (n = 14)	Without HCQ/CQ (n = 21)	*P* value
Age	62.20 ± 11.88		61.00 ± 13.00	63.00 ± 11.33	0.633
Male	23		10	13	0.721
Disease duration (days)	13.00 ± 7.24		13.00 ± 7.14	13.00 ± 7.49	1.000
Clinical manifestation at beginning					
Fever	27		10	17	0.685
Fatigue	25		11	14	0.704
Cough	26		12	14	0.262
Diarrhea	8		3	5	1.000
Myalgia/arthralgia	10		7	3	0.053
Fever at admission	7		2	5	0.676
Comorbidities					
Hypertension	13		7	6	0.199
Diabetes mellites	5		2	3	1.000
Carcinoma^a^	5		3	2	0.369
Stroke	1		1	0	0.400
Coronary artery disease	2		1	1	1.000
Lung disease^b^	6		1	5	0.366
HBV infection	7		3	4	1.000
Disease severity status					
General	19		7	12	0.678
Severe/critical	16		7	9	
CURB-65 score					
0	15		6	9	1.000
1–5	20		8	12	
Laboratory results					
WBC (× 10^9^/L)	6.13 ± 2.45		6.27 ± 2.96	6.05 ± 2.12	0.801
Neu (× 10^9^/L)	4.33 ± 2.38		4.68 ± 2.88	4.10 ± 2.01	0.490
Lym (× 10^9^/L)	1.08 ± 0.52		1.05 ± 0.56	1.09 ± 0.50	0.828
Neu/Lym	5.27 ± 4.31		6.16 ± 5.13	4.69 ± 3.67	0.329
Hb (g/L)	123.17 ± 18.43		126.14 ± 19.22	121.19 ± 18.08	0.444
PLT (× 10^9^/L)	266.37 ± 111.96		283.86 ± 110.58	254.71 ± 114.04	0.459
ALT (U/L)	33.14 ± 28.69		27.00 ± 21.31	37.24 ± 32.56	0.308
AST (U/L)	32.11 ± 20.96		27.00 ± 13.72	35.52 ± 24.37	0.244
Alb (g/L)	33.26 ± 5.60		31.94 ± 6.51	34.14 ± 4.87	0.260
LDH (U/L)	282.69 ± 126.59		310.00 ± 130.40	264.48 ± 123.78	0.304
eGFR (mL/min/1.73 m^2^)	89.33 ± 15.88		86.17 ± 12.15	91.43 ± 17.92	0.345
> 90 mL/min/1.73 m^2^	20		7	13	0.486
≤ 90 mL/min/1.73 m^2^	15		7	8	
Fibrinogen (g/L)	5.17 ± 1.59		5.82 ± 1.19	4.74 ± 1.66	0.045
d-Dimer (ug/mL FEU)	3.63 ± 5.42		4.30 ± 6.65	3.18 ± 4.54	0.556
> 1.0 ug/mL FEU	21		8	13	0.778
≤ 1.0 ug/mL FEU	14		6	8	
NT-pro-BNP (ug/mL)	253.20 ± 346.51		318.29 ± 520.19	209.81 ± 152.60	0.372
cTnI (pg/mL)	9.11 ± 9.51		8.43 ± 9.38	9.56 ± 9.79	0.736
ESR (mm/h) (/n)	47.75 ± 26.74 (32)		58.62 ± 19.90 (13)	40.32 ± 28.70 (19)	0.056
hsCRP (mg/L)	33.89 ± 38.61		31.47 ± 24.06	35.50 ± 46.39	0.767
Procalcitonin (ng/mL)	0.17 ± 0.46		0.10 ± 0.08	0.21 ± 0.60	0.507
≥ 0.1 ng/mL	9		5	4	0.432
< 0.1 ng/mL	26		9	17	
Ferritin (ug/L) (n)	819.36 ± 628.02 (31)		689.45 ± 494.53 (13)	913.18 ± 707.92 (18)	0.336
IL-6 (ug/mL) (n)	14.49 ± 15.62 (31)		13.28 ± 9.27 (13)	15.37 ± 19.19 (18)	0.721
Other respiratory pathogen infection^c^	21		10	11	0.260
Imaging findings					
GGO	30		14	16	0.069
Consolidation	19		7	12	0.678
Bilateral pulmonary infiltration	34		14	20	1.000
Interstitial changes	17		7	10	0.890
Hydrothorax	7		1	6	0.203

*HBV* hepatitis B virus, *WBC* white blood cell, *Neu* neutrophil, *Lym* lymphocyte, *Hb* hemoglobulin, *PLT* platelet, *ALT* alanine transaminase, *AST* oxaloacetic transaminase, *LDH* lactate dehydrogenase, *eGFR* estimated glomerular filter rate, *NT-pro-BNP* N-terminal pro-Brain Natriuretic Peptide, *cTnI* cardiac troponin I, *ESR* erythrocyte sedimentation rate, *hsCRP* high sensitivity C reactive protein, *IL-6* interleukin-6, *GGO* ground glass opacity, *HCQ* hydroxychloroquine, *CQ* chloroquine

^a^Including carcinoma in the stomach (n = 2), urinary bladder (n = 1), bone (n = 1) and breast (n = 1)

^b^Lung disease refers to chronic obstructive lung disease (n = 3), emphysema (n = 2), bronchiectasis (n = 1), lung fibrosis (n = 1) and bullae (n = 1)

^c^Other concurrent respiratory pathogen infection with a specific serum immunoglobulin M positive confirmed by the enzyme-linked immunosorbent assay includes type A influenza (n = 18), type B influenza (n = 2), mycoplasma pneumoniae (n = 2) and chlamydia pneumoniae (n = 1)

Twenty-two patients took anti-influenza drugs, i.e., oseltamivir or arbidol or both. Most patients (94.28%) received traditional Chinese medicine (TCM) treatment. And the total types of anti-virus agents were similar between these two treatment groups (Table 2). Antibiotics were concomitantly administrated with HCQ/CQ in 17 patients. And moxifloxacin was the most commonly used antibiotic (13/17). HCQ/CQ was not administrated in combination with azithromycin in our patients. GCs were administrated in 12 (34.28%) patients. There were more patients taking GCs in the HCQ/CQ treatment group (57.14% vs. 19.05%, *P* = 0.031) (Table 2). The detailed information of GCs was available in 11 patients. Patients took GCs at a median of 14 days after symptoms onset (Q1: 12 days, Q3: 19 days). The GCs treatment lasted for a median of 6 days (Q1: 4 days, Q3: 7 days). And the median cumulated dosage of GCs was 280 mg (MP or equivalent, Q1: 160 mg, Q3: 480 mg).

**Table 2:** Treatment and outcomes of the 35 patients

	Total (n = 35)	With HCQ/CQ (n = 14)	Without HCQ/CQ (n = 21)	*P* value
Treatment				
Antivirus agents				
Ribavirin	9	4	5	1.000
Lopinavir/Ritonavir	4	1	3	0.635
Oseltamivir	18	8	10	0.581
Arbidol	10	6	4	0.151
TCM	33	14	19	0.506
Types of antivirus agents	2.11 ± 0.93	2.36 ± 0.75	1.95 ± 1.02	0.213
Corticosteroids	12	8	4	0.031
IVIG	9	4	5	1.000
Antibiotics	22	10	12	0.392
Anticoagulant	8	3	5	1.000
Virus shedding period (days)	22.09 ± 9.51	26.57 ± 10.35	19.10 ± 7.80	0.020
Swab testing times	3.81 ± 2.04	5.15 ± 2.38	2.89 ± 1.10	0.001
Consecutive swab testing negative times before discharging	3.03 ± 1.23	3.23 ± 1.42	2.89 ± 1.10	0.457
Swab testing interval (days)	6.10 ± 1.63	5.77 ± 1.36	6.34 ± 1.80	0.346
Outcomings				
Discharged	32	12	20	0.551
Deceased	3	2	1	

*TCM* traditional Chinese medicine, *IVIG* intravenous immune globulin, *HCQ* hydroxychloroquine, *CQ* chloroquine

**Fig. 2:**
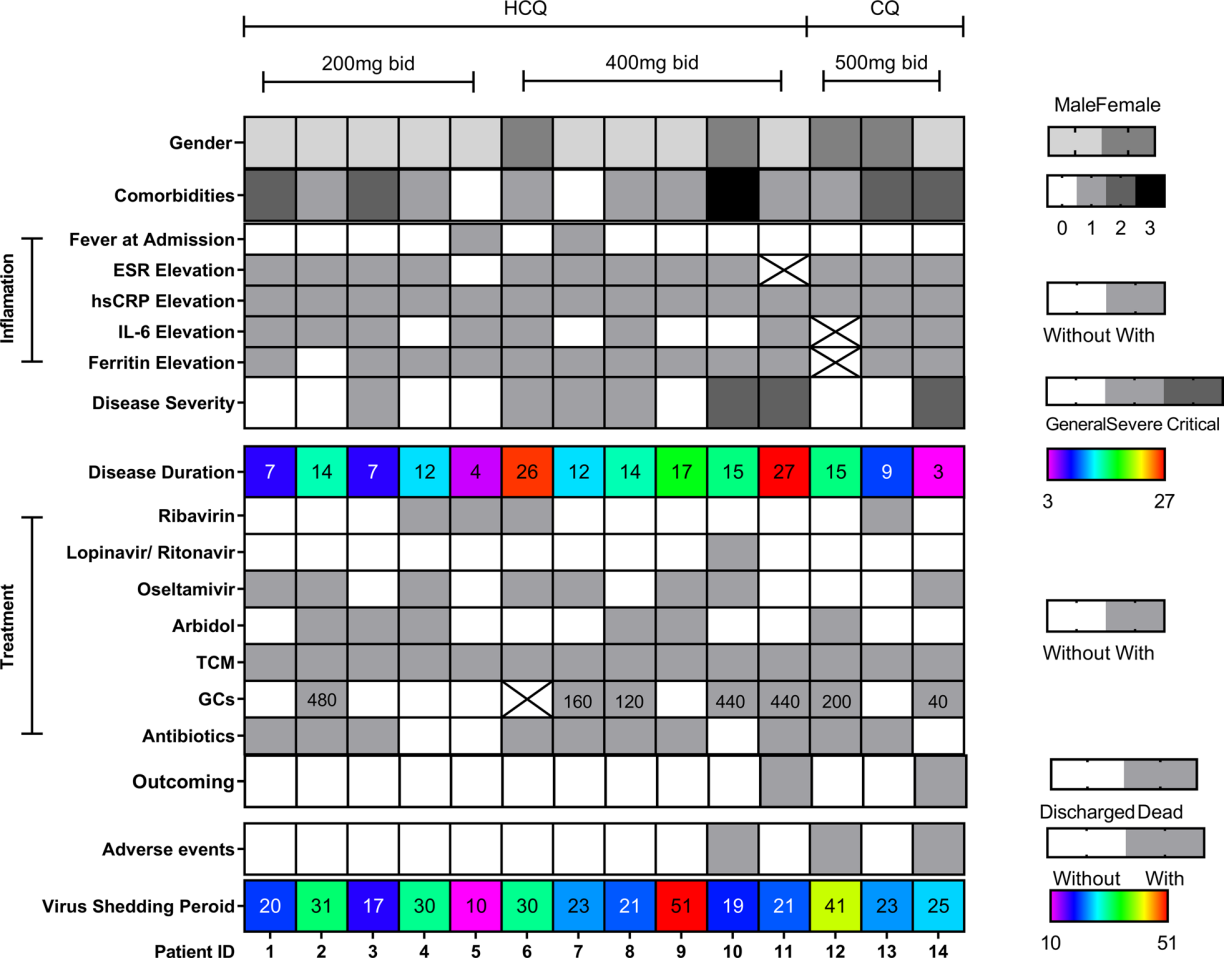
The detailed clinical, treatment and outcome information of patients with HCQ/CQ treatment. *HCQ* hydroxychloroquine, *CQ* chloroquine, *ESR* erythrocyte sedimentation rate, *hsCRP* high sensitivity C reactive protein, *IL-6* interleukin-6, *TCM* traditional Chinese Medicine, *GCs* glucocorticoids. Comorbidities refers to the types of comorbidities in one single patient. Disease severity was classified according to the Chinese management guideline for COVID-19. Disease duration (days) was calculated from symptom onset to inpatient department admission. GCs were summed as methylprednisolone or equivalent and the total dosages of GCs were recorded in the corresponding square, respectively. Patient No.10 had transient first-degree atrioventricular block (AVB). Patient No.12 complained about dizziness and blurred vision which disappeared after CQ discontinuation. Patient No.14 had recurrent AVB and obvious QTc elongation even after CQ withdrawn. The virus shedding period was defined from symptoms onset to the first day of the consecutive negative PCR results before discharge. The X was put in the square in which the data was not available

**Fig. 3:**
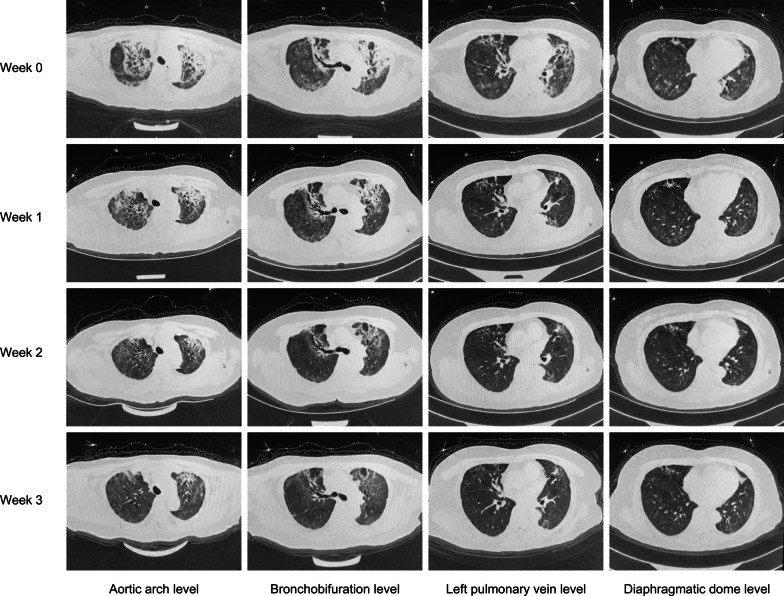
The computed tomography findings of one patient (No. 6) before (week 0) and 1, 2, 3 weeks after HCQ administration, respectively. After the comprehensive treatment together with HCQ, the ground-glass opacity lesions were largely absorbed, while some of the fibrosis stripe lesions were left

The dosage of HCQ was either 200 mg (n = 5) or 400 mg (n = 6) twice a day. And the dosage of CQ was 500 mg (n = 3) twice a day (Fig. 2). The average disease duration before HCQ/CQ initiation was 21.00 ± 5.98 days (Q1: 16.50 days; Q2: 22.00 days; Q3: 26.25 days). The HCQ/CQ treatment lasted for an average of 10.36 ± 3.12 days (Q1: 10.75 days; Q2: 11.00 days; Q3: 12.00 days). Only 1 of the 14 patients received HCQ/CQ treatment after virus shedding. The SARS-Cov-2 RNA tests turned negative after an average of 7.31 ± 6.05 days (Q1: 3.00 days; Q2: 5.00 days; Q3: 9.50 days) since HCQ/CQ initiation in the rest 13 patients. The average VSPs were 22.09 ± 9.51 days, which was a little longer in the HCQ/CQ treatment group (26.57 ± 10.35 days vs. 19.10 ± 7.80 days, *P* = 0.020). However, the average swab testing intervals didn’t differ between patients with and without HCQ/CQ treatment statistically (5.77 ± 1.36 days vs. 6.34 ± 1.80 days, *P* = 0.346) (Table 2). For the patients whose VSPs were longer than 22 days, the differences of average VSPs in patients with and without HCQ/CQ treatment were not statistically different (31.75 ± 9.72 days/n = 8 vs. 28.67 ± 3.56 days/n = 6, *P* = 0.477). In the multivariate linear regression analysis, disease durations at admission (t = 3.643, *P* = 0.001) and HCQ/CQ treatment (t = 2.637, *P* = 0.013) were independent predict parameters for patients’ VSPs prediction (Additional file 1: Table S1). The linear regression formulation was listed as following. Here is an example. One male patient with COVID-19 was admitted to the hospital at the 5th day after symptom onset. He received HCQ therapy in the hospital. Therefore his expected virus shedding period was 21 (10.039 + 0.697 × 5 + 7.140 × 1 = 20.664) days. Meanwhile, neither GCs treatment (t = − 0.313, *P* = 0.772) nor GCs dosage (t = − 0.706, *P* = 0.766) was related to VSPs statistically. And after treatment, acute exudation lesions were largely absorbed in pulmonary CT (Fig. 3). There were 3 patients deceased during inpatient period in our study, and two patients were with HCQ/CQ treatment (*P* = 0.551). Two patients died from multiple organ failure. And the other patient died suddenly. Their relatives refused of autopsy. Thus, the exact reasons for their death were unknown.

Virus shedding period (days) = 10.039 + 0.697 × disease durations at admission + 7.140 × with or without HCQ/CQ treatment (0, if without HCQ/CQ treatment; 1, if with HCQ/CQ treatment).

Electrocardiographs (ECGs) were conducted at least once in 12 out of the 14 patients (9 patients with HCQ treatment, and 3 patients with CQ treatment). First-degree atrioventricular block (AVB) was recorded in 2 patients. One patient received HCQ and the other received CQ. No second or third AVB was noticed. First-degree AVB disappeared after HCQ discontinuation. However, the first-degree AVB disappeared after CQ discontinuation and reoccurred 10 days later. The QTc interval longer than 500 ms was recorded in the identical patient with CQ treatment. Another patient with CQ treatment complained about dizziness and blurred vision. And the symptoms disappeared after CQ being withdrawn. No patient complained about new symptoms during HCQ treatment.

## Discussion

The conventional anti-malaria drug HCQ/CQ was regarded as a promising agent for its dual effects on inflammation modulation and virus inhibition since the beginning of the pandemic [12, 14, 15].

During the past decades, several researchers had confirmed the anti-virus effects of HCQ/CQ in vitro and in vivo [7, 9, 10]. HCQ/CQ could prevent the coronavirus from entering the host cells by interfering with endosomal acidification which is essential for membrane fusion. However, coronavirus could invade the host cells via alternative non-endosomal pathway which is not blocked by HCQ/CQ [20]. CQ could also interfere with virus post translation modification by pondus hydrogenii (PH) modulation [21]. At the meantime, HCQ/CQ could act on host cells directly. HCQ/CQ could inhibit glycosylation of the cell membrane protein angiotensin converting enzyme-2, to which the SARS-Cov-2 is attached [22]. HCQ/CQ could downregulate the toll like receptor (TLR) on activated immune cells and block TLR signal transduction, and prohibit inflammatory factors secretion, such as IL-6 [8, 23].

By far, a few clinical studies have analyzed the efficacy of HCQ/CQ in COVID-19. Gautret and colleagues reported that most patients with COVID-19 were virologicaly cured 6 days after HCQ initiation, especially those who received HCQ in combination with azithromycin [24]. However, Gautret et al.’s study had a relatively small sample size and two selection bias. First, patients in the treatment and control group were not from the same medical center. Second, the virus loads in the HCQ treatment group were lower compared to those in the control group at inclusion. Lower virus loads indicated that the patients in the HCQ treatment group were at a later disease phase of SARS-Cov-2 infection and were more likely to have autolimiting disease course [25]. In a randomized clinical trial (RCT), Chen and colleagues reported that after HCQ treatment with a dosage of 400 mg/day for 5 days, the clinical and radiological improve rates were higher compared to those in patients without HCQ treatment (80.6% vs. 54.8%) [26]. In another randomized study with mildly to moderately ill COVID-19 patients, Tang et al*.* noticed that the SARS-Cov-2 negative conversion rates were similar in patients with and without HCQ treatment (85.4% vs. 81.3%) [27]. In a retrospective study, Mallat and colleagues reported that HCQ treatment was an independent factor for longer VSPs. The median time span from nasopharyngeal swab positivity to negativity were 17 days in the HCQ treatment group and 10 days in the control group, respectively (*P* = 0.023). HCQ was administrated at an early stage of the disease course in Mallat’s study [28].

In our study, the number of mortality cases were not statistically different between patients with and without HCQ/CQ treatment. The result might be ascribed to several factors. Firstly, HCQ/CQ was administrated at a later phase of the disease course. In some patients, we used HCQ/CQ due to persistent SARS-Cov-2 RNA positivity for salvage treatment purposes. It is widely accepted that anti-virus should be taken as early as possible in influenza and corona virus infection [4, 29]. Secondly, the half-life of HCQ/CQ is as long as 40–60 days due to the large distribution volume in the blood. And it usually takes several weeks before HCQ/CQ reaching its maximal activity [30]. In COVID-19, HCQ/CQ treatment only lasted for an average of 10 days. Therefore, HCQ/CQ might be withdrawn before it worked. Thirdly, for ethic factors concern, several kinds of drugs, such as GCs, ribavirin, TCM et al*.*, were administrated empirically and anecdotally at the same time. These concomitantly taken drugs might have covered up the potential therapeutic effects of HCQ/CQ on COVID-19. Fourthly, due to the small sample size, the death rates were not statistically different in patients with and without HCQ/CQ treatment. Taken together, the efficacy of HCQ/CQ in COVID-19 management should be verified in large randomized controlled trials.

In the present study, the average VSPs were similar to those reported in the previous study [31]. After the multivariate linear regression analysis, we identified that disease durations at admission and HCQ/CQ treatment were independent parameters related to patients’ VSPs, indicating patients might have better prognosis if being well treated earlier. Furthermore, VSPs were not statistically different between patients with longer VSPs (VSPs > 22 days) in these two treatment groups. It was interesting that there were more patients who took GCs in the HCQ/CQ treatment group. However, after being adjusted by other confounders, neither GCs treatment nor GCs dosage was an independent parameter for VSPs prediction. Actually, the effect of GCs on COVID-19 remains controversial and disputable. In SARS and Middle East Respiratory Syndrome (MERS), GCs administration was related to delayed virus RNA clearance [32, 33]. However, in the SARS or MERS studies, patients were either critically ill [33] or took rather high GCs dosage [32]. On the other hand, patients with SARS or influenza might benefit from low-to-moderate GCs [34, 35]. In the present study, our patients took a low-to-moderate dose of GCs during a relative short period of time. As a result, we didn’t find correlations between GCs treatment and prolonged VSPs. A team consist of front-line physicians from the Chinese Thoracic Society suggested that after careful benefits and harms evaluation, short term low-to-moderate dose of GCs could be prudently administrated in patients with COVID-19 [36].

One of the major concerns for HCQ/CQ treatment in COVID-19 is the side effect [37]. HCQ/CQ related retinopathy always occurs after months even years of HCQ/CQ administration [30]. Meanwhile, HCQ/CQ related arrythmia might be lethal. And the risk is rising together with other arrhythmogenic drugs, such as azithromycin [30]. Borba et al. reported that high dose of CQ (600 mg twice daily) was related to prolonged QTc interval and should not be recommended in critically ill patients [38]. Lane and colleagues reported that HCQ monotherapy was safe in COVID-19. However, HCQ in addition to azithromycin might result in heart failure and cardiovascular mortality [39]. Tang et al*.* found that HCQ was safe in patients with COVID-19, the most common adverse effects were diarrhea and vomiting [27]. Similarly, HCQ was safe and tolerable in our patients. On the contrast, among the three patients with CQ treatment, one patient complained about dizziness and blurred vision and another patient had recurrent first-degree AVB and obvious QTc elongation.

The major limitation of the study was the relatively small sample size. There were only 14 patients received HCQ/CQ treatment due to the unsettled debate on the safety profile of HCQ/CQ in COVID-19. The sample size of the patients without HCQ/CQ was expected to be 28. However, after age, gender and disease severity matching, only 21 patients without HCQ/CQ treatment met the matching criteria and were finally selected. Secondly, some patients were treated with HCQ/CQ for persistent SARS-Cov-2 RNA positivity. These patients, per se, were refractory to treatment. Therefore, selection bias exists in our patients. Thirdly, due to the retrospective nature of the study, although we found out that HCQ/CQ treatment was related to longer VSPs, we couldn’t tell whether HCQ/CQ prolonged SARS-Cov-2 RNA clearance or not.

## Conclusions

In summary, we identify that the HCQ/CQ administration is not related to neither less mortality cases nor shorter VSPs at later phase of COVID-19. More studies are needed to explore whether HCQ/CQ treatment would lead to SARS-Cov-2 RNA clearance delay or not. And HCQ other than CQ is a safe and tolerable drug in COVID-19 patients.

## Supplementary Information

**Additional file 1.** The disease severity definition and discharging criteria according to the Chinese management guideline for COVID-19. **Table S1.** The details of model for VSPs prediction.

## Data Availability

The datasets used and/or analyzed during the current study are available from the corresponding author on reasonable request.

## Abbreviations

HCQ: Hydroxychloroquine
CQ: Chloroquine
SARS-Cov-2: Severe acute respiratory syndrome coronavirus 2
COVID-19: Corona virus disease-2019
SLE: Systemic lupus erythematosus
IL-6: Interleukin-6
CT: Computed tomography
eGFR: Estimated glomerular filtration rate
RNAs: Ribonucleic acids
PCR: Polymerase chain reaction
VSPs: Virus shedding periods
GCs: Corticosteroids
MP: Methylprednisolone
SD: Standard deviation
TNF-α: Tumor necrosis factor-α
TCM: Traditional Chinese medicine
ECGs: Electrocardiographs
AVB: Atrioventricular block
TLR: Toll like receptor
RCT: Randomized clinical trial
MERS: Middle East Respiratory Syndrome
