# Supplementary material for: Hydroxychloroquine/chloroquine in patients with COVID-19 in Wuhan, China: a retrospective cohort study
Source: BMC Infect Dis. 2021 Aug 12;21:805. doi: 10.1186/s12879-021-06477-x (PMC8358550; doi:10.1186/s12879-021-06477-x)
Supplement: Supplementary file 1 — Additional file 1. The disease severity definition and discharging criteria according to the Chinese management guideline for COVID-19. Table S1. The details of model for VSPs prediction. [file 12879_2021_6477_MOESM1_ESM.doc]

Additional file 1. The disease severity definition and discharging criteria according to the Chinese management guideline for COVID-19.

**Disease severity definition:**

Mild was defined as mild symptoms without radiology confirmed pneumonia; general was defined as fever or respiratory system symptoms with radiology confirmed pneumonia; severe was defined as respiratory rate≥ 30 per minute or peripheral oxygen saturation rate≤ 93%or arterial oxygen pressure (PaO_2_)/ fraction of inspiration oxygen (FiO_2_)≤ 300mmHg(1mmHg=0.133kPa) adjusted by altitude or radiology confirmed pulmonary lesions progressing by more than 50% within 24-48 hours; critically severe was defined as respiratory failure requiring mechanical ventilation or shock or multiple organ failure requiring close monitoring in the intensive care unit[1].

**Discharging criteria:**

The patients would not be discharged until all the 4 criteria was met. 1) the temperature remained normal for at least 3 consecutive days; 2) respiratory symptoms ameliorated; 3) acute exudation lesions were largely absorbed assessed by pulmonary CT; and 4) two consecutive results of undetectable SARS-Cov-2 RNAs by swabs via PCR were recorded (at least 24 hours apart) [1].

Table S1. The details of model for VSPs prediction.

Table S1. The coding, β score, t score and *P* value of probable predict variables in the model.

|  | Coding | β scores | t scores | *P* values |
| --- | --- | --- | --- | --- |
| Dependent variable |  |  |  |  |
| Virus shedding periods (days) | Continuous (days) | / | / | */* |
| Predict variables |  |  |  |  |
| (Constant) | Continuous (days) | ***10.039*** | ***3.352*** | ***0.002*** |
| Age | Continuous (years) | -0.017 | -0.115 | 0.909 |
| Male | Binary (no 0, yes 1) | 0.046 | 0.316 | 0.754 |
| Disease duration at admission | Continuous (days) | ***0.697*** | ***3.643*** | ***0.001*** |
| Hypertension | Binary (no 0, yes 1) | 0.066 | 0.444 | 0.661 |
| Diabetes mellites | Binary (no 0, yes 1) | -0.125 | -0.873 | 0.389 |
| Carcinoma* | Binary (no 0, yes 1) | -0.088 | -0.606 | 0.549 |
| Stroke | Binary (no 0, yes 1) | -0.171 | -1.185 | 0.245 |
| Coronary artery disease | Binary (no 0, yes 1) | -0.089 | -0.607 | 0.548 |
| Lung disease** | Binary (no 0, yes 1) | 0.098 | 0.665 | 0.511 |
| HBV infection | Binary (no 0, yes 1) | 0.215 | 1.517 | 0.140 |
| Lymphocyte | Continuous (x10^9^/ L) | -0.048 | -0.328 | 0.746 |
| Neutrophil/ Lymphocyte | Continuous | -0.190 | -1.320 | 0.197 |
| Fibrinogen | Continuous (g/L) | 0.169 | 1.118 | 0.272 |
| Ribavirin | Binary (no 0, yes 1) | 0.029 | 0.203 | 0.841 |
| Lopinavir/ Ritonavir | Binary (no 0, yes 1) | 0.110 | 0.763 | 0.452 |
| Oseltamivir | Binary (no 0, yes 1) | 0.201 | 1.439 | 0.160 |
| Arbidol | Binary (no 0, yes 1) | 0.192 | 1.316 | 0.198 |
| TCM | Binary (no 0, yes 1) | 0.123 | 0.846 | 0.404 |
| HCQ/CQ | Binary (no 0, yes 1) | ***7.140*** | ***2.637*** | ***0.013*** |
| IVIG | Binary (no 0, yes 1) | 0.058 | 0.400 | 0.692 |
| Antibiotics | Binary (no 0, yes 1) | 0.198 | 1.413 | 0.168 |
| Anticoagulant | Binary (no 0, yes 1) | -0.074 | -0.512 | 0.612 |
| Corticosteroids | Binary (no 0, yes 1) | -0.051 | -0.313 | 0.756 |
| Cumulated corticosteroid dosages | Continuous (mg) | -0.115 | -0.706 | 0.486 |

* Including carcinoma in the stomach (n=2), urinary bladder (n=1), bone (n=1) and breast (n=1); ** lung disease refers to chronic obstructive lung disease (n=3), emphysema (n=2), bronchiectasis (n=1), lung fibrosis (n=1) and bullae (n=1); HBV: hepatitis B virus; TCM: traditional Chinese medicine; HCQ: hydroxychloroquine; CQ: chloroquine; IVIG: intravenous immune globulin.

**References**
